# Activated Carbon from Pyrolysis of Plastic Waste as an Adsorbent for the Removal of Pb(II), Cd(II) and Co(II) from Aqueous Solutions

**DOI:** 10.3390/ma19163522

**Published:** 2026-08-19

**Authors:** Beata Jabłońska, Gabriela Poznańska, Paweł Jabłoński, Jerzy Gęga

**Affiliations:** 1 Department of Environmental Engineering and Biotechnology, Faculty of Infrastructure and Environment, Czestochowa University of Technology, Brzeźnicka St. 60A, 42-201 Częstochowa, Poland; 2 HIG Polska Sp. z o.o., Niedźwiedziniec St. 18, 41-506 Chorzów, Poland; gabriela.poznanska@higpolska.pl; 3 Faculty of Electrical Engineering, Czestochowa University of Technology, 17 Armii Krajowej Ave., 42-201 Częstochowa, Poland; pawel.jablonski@pcz.pl; 4 Department of Materials Science and Engineering, Faculty of Production Engineering and Materials Technology, Czestochowa University of Technology, Armii Krajowej 19, 42-201 Częstochowa, Poland; jerzy.gega@pcz.pl

**Keywords:** char, post-pyrolysis char, plastic waste, adsorption, activated carbon, heavy metals

## Abstract

**Highlights:**

- Application of post-pyrolysis char from plastic wastes.
- Chemical activation of the char produces an activated carbon.
- The activated carbon can be used to adsorb Pb(II), Cd(II) and Co(II) ions.
- A new direction of plastic waste management.
- Post-pyrolysis char after activation can be used in water treatment processes.

**Abstract:**

Slow pyrolysis of a plastic fraction isolated from municipal waste produced a char, which was then used as a precursor for the synthesis of activated carbon. The process involved thermal conversion at 800 °C and chemical activation using K_2_CO_3_. The resulting activated carbon was used to remove Pb(II), Cd(II), and Co(II) from aqueous solutions. Physicochemical, structural, and granulometric characterizations of the resulting adsorbent were performed. The obtained material had a specific surface area of 562 m^2^/g, a total pore volume of 0.328 cm^3^/g, and a micropore volume of 0.146 cm^3^/g. To determine the optimal adsorption conditions, the Box–Behnken experiment planning method was used, assuming solution pH, adsorbent mass, and initial metal ion concentration as independent variables, and the percentage removal of the contaminant as the response. Studies on sorption isotherms were conducted using a static method in a periodic system for initial metal ion concentrations ranging from 10 to 250 mg/dm^3^. The effect of temperature on the adsorption process was analyzed, and the kinetics sorption was investigated. Several adsorption isotherm models were used to describe the adsorption equilibrium. The maximum sorption capacity was 35.5 mg/g for Pb(II), 14.7 mg/g for Cd(II), and 11.6 mg/g for Co(II). The obtained results indicate that the plastic waste based adsorbent exhibits favorable sorption properties for the tested heavy metal ions and may be useful in water and wastewater treatment processes.

## 1. Introduction

Currently, plastic pollution is one of the greatest challenges in environmental protection. Recent years have seen a dynamic increase in global plastics production, which increased from 370.6 million Mg in 2018 to 430.9 million Mg in 2024 [1]. At the same time, the importance of chemical recycling as a method for managing polymer waste is constantly growing, especially in the case of plastics with a heterogeneous composition and a high degree of contamination with technological additives, such as plasticizers, dyes, or stabilizers [2,3,4]. These materials often do not meet the quality requirements necessary for effective processing using mechanical recycling methods, which limits their reuse possibilities. Among the available chemical recycling technologies, a particularly promising method is pyrolysis, which involves the thermal decomposition of polymeric materials in conditions of limited oxygen or complete absence of oxygen [5,6,7]. According to [8], the conversion of plastic waste by pyrolysis is one of the most effective chemical recycling methods, enabling both the recovery of valuable products and a significant reduction in the amount of waste deposited in the environment. The process results in three main fractions, gaseous, liquid, and solid (char), the quantity, composition, and properties of which depend on the type of material processed and the process parameters [2,9,10]. Numerous studies have shown that the highest efficiency of the solid fraction is obtained during slow pyrolysis [11]. This process is characterized by a prolonged residence time of the batch in the reactor compared to fast pyrolysis [12]. Slow pyrolysis is typically conducted at temperatures of 300–600 °C, with a low heating rate (below 5 °C/min) and a long material retention time in the reactor (1–5 h). Such conditions favor secondary transformations of primary products, leading to increased char yield at the expense of gaseous and liquid fractions [13]. However, the importance of pyrolysis goes beyond the mere reduction in polymer waste. This technology enables the effective management of mixed plastic waste, which is difficult to process using mechanical recycling methods. At the same time, it contributes to the reduction in greenhouse gas emissions and the recovery of materials and energy, in line with the assumptions of a circular economy [14]. A particularly important product in the process is char, which, after appropriate physical or chemical activation, can be a valuable precursor for the production of carbon materials with a high specific surface area, favorable porous structure, and diverse surface chemistry [15,16,17]. These properties determine a wide range of applications, especially as adsorbents in water and wastewater treatment processes. Activated carbons obtained from plastic waste are an attractive alternative to conventional adsorbents produced from virgin raw materials. Their use is consistent with the principles of a circular economy, enabling the simultaneous management of problematic waste streams and the production of materials with high utility value [18]. Due to their developed porous structure and the presence of functional groups on the surface, these materials may exhibit an affinity for many pollutants present in the aquatic environment, including heavy metal ions [14,19].

Water pollution with heavy metals is one of the most serious environmental problems in the modern world [20]. Metal ions such as Pb(II), Cd(II), and Co(II) can enter the aquatic environment along with wastewater from the metallurgical, electroplating, mining, chemical, and battery industries [20,21]. Due to their persistence, bioaccumulation capacity, and potential toxic effects even at low concentrations, these metals pose a significant threat to both aquatic ecosystems and human health [22,23]. Lead and cadmium are particularly hazardous elements, exhibiting neurotoxic, nephrotoxic, and carcinogenic properties [20,24,25]. Cobalt is a trace element essential for the proper functioning of living organisms, but its excessive concentration can cause metabolic disorders, oxidative stress, and negatively impact aquatic organisms [26]. Due to growing anthropogenic pressure and increasing emissions of heavy metals into the environment, the development of effective methods for their removal from aqueous solutions remains one of the key challenges of modern environmental engineering.

Although interest in the use of activated carbons obtained from plastic waste has significantly increased, the number of studies on their application in the removal of heavy metals from aqueous solutions remains limited. Previous work has focused mainly on the physicochemical characteristics of the obtained materials and the adsorption of selected organic pollutants, while issues related to the removal of heavy metal ions, especially Co(II), remain relatively poorly understood [18,27,28]. Furthermore, the influence of the surface properties of adsorbents obtained from plastic waste on the binding efficiency of individual metal ions remains insufficiently understood.

Therefore, the aim of this study was to determine the potential use of char produced during the pyrolysis of mixed plastic waste as a precursor for the production of activated carbon and to assess its suitability as an adsorbent for the removal of Pb(II), Cd(II), and Co(II) ions from aqueous solutions. In contrast to most studies in the literature, which employ single and well-defined polymer wastes, e.g., poly(ethylene terephthalate) (PET), polypropylene (PP) or polyethylene (PE), the precursor used in this study was obtained from the plastic fraction separated from municipal solid waste, representing a realistic and heterogeneous waste stream generated in municipal waste management. This study characterized the physicochemical properties of the obtained material and determined its sorption capacity for selected heavy metals. The obtained results allow for an assessment of the potential of carbonaceous materials derived from recycled plastic waste as effective adsorbents used in water and wastewater treatment processes.

## 2. Materials and Methods

### 2.1. Materials

#### 2.1.1. Char

**Figure 1:**
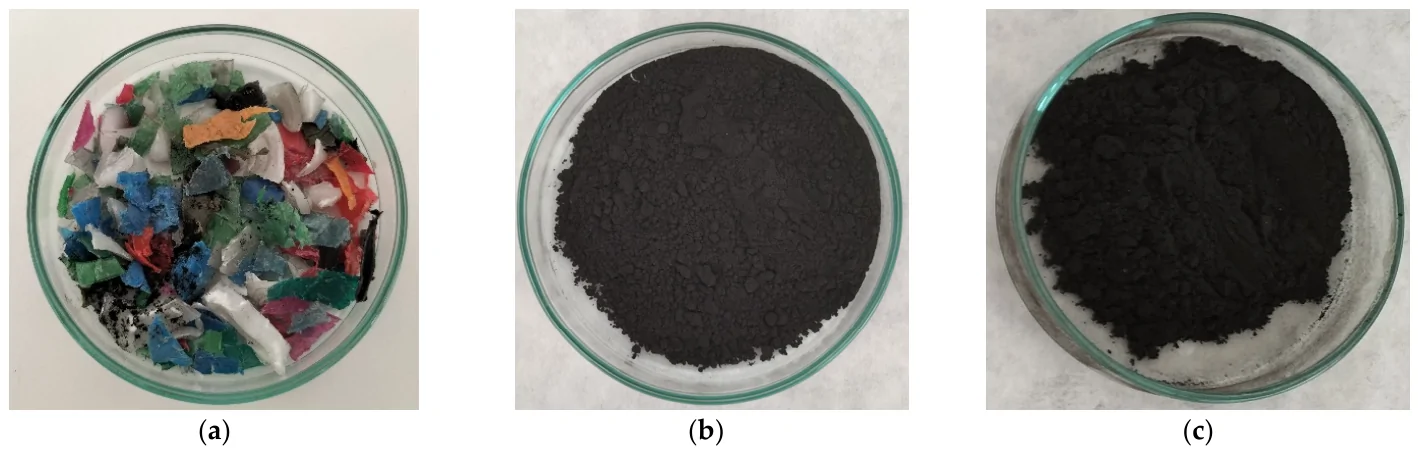
View of the material used in the research: (**a**) polymer municipal waste; (**b**) post-pyrolysis char; (**c**) activated carbon after thermochemical modification.

The adsorption process used char obtained from selected municipal waste, constituting the polyolefin fraction of packaging waste (Figure 1a). The polymer waste came from the sorting process of municipal waste and included a mixture of polyolefins—mainly PE (approx. 60%, including LDPE of approx. 30% and HDPE of approx. 30%) and PP (approx. 40%), with code ex 15 01 02. The char was obtained from HIG Polska (Chorzów, Poland) specializing in the processing of polymer waste in chemical recycling processes (Figure 1b).

#### 2.1.2. Preparation of Activated Carbon

The char was chemically activated using potassium carbonate (K_2_CO_3_). For this purpose, the char and K_2_CO_3_ were mixed in a 1:1 mass ratio. The prepared mixture was placed in a reactor and pyrolyzed in a nitrogen stream (5 dm^3^/min). The temperature was gradually increased at a rate of 10 °C/min to 800 °C and then maintained for 2 h. After the process was completed, the samples were left in the reactor to cool naturally to room temperature. Pyrolysis was carried out in a horizontal tube furnace (PRW-S100 × 780/11 manufactured by Czylok Sp. z o. o., Jastrzębie Zdrój, Poland). The obtained material was washed with 5% HCl solution and left for 24 h. Then, the sample was intensively rinsed with hot distilled water until the pH in the filtrate was around 7, after which it was dried at 105 °C until a constant mass was obtained. The activated carbon obtained in this way (Figure 1c) was stored in a glass container and marked as PWAC (Polymer Waste-based Activated Carbon).

#### 2.1.3. Heavy Metal Reagents

Solutions of heavy metal ions Pb(II), Cd(II) and Co(II) were prepared from lead(II) nitrate (Pb(NO_3_)_2_, analytical grade), cadmium(II) nitrate tetrahydrate (Cd(NO_3_)_2_ 4H_2_O) and cobalt(II) nitrate hexahydrate (Co(NO_3_)_2_ 6H_2_O), respectively, purchased from Pol-Aura Odczynniki Chemiczne. These metals were selected for sorption studies due to their common occurrence in industrial wastewater and polluted waters and their potentially harmful effects on the environment and living organisms [29,30,31]. Additionally, these metals differ in their chemical properties, which allows for the assessment of the effectiveness of the tested process in removing metal ions with different characteristics.

### 2.2. Methods

#### 2.2.1. Physicochemical Analyses of Activated Carbon

The physicochemical properties of the obtained activated carbon, including pH, moisture content, loss on ignition, ash content, volatile substances, and fixed carbon, were determined in accordance with applicable standards. The pH value was determined potentiometrically using a pH meter. Total moisture content was determined gravimetrically in accordance with PN-EN ISO 18134-2:2017-03 [32]. Ash content was determined gravimetrically in accordance with PN-ISO 1171:2002 [33]; the samples were ashed and calcined to constant weight at 815 ± 10 °C for two hours in an FCF22S muffle furnace (CZYLOK, Jastrzębie Zdrój, Poland). The content of combustible components (loss of ignition) was calculated relative to the dry mass of char using the following formula:

(1) LOI=100−A

where A is the content of non-combustible components (ash). The content of volatile matter (VM) was determined in accordance with PN-EN ISO 18123:2016-01 [34]. The method involves calcining a sample of the tested material at a temperature of 815 ± 10 °C under anaerobic conditions for 7 min. The content of carbon bound to solid matter (fixed carbon) was calculated as follows:

(2) FC=100−VM−A

where FC is the fixed carbon content in the sample on a dry basis in %; VM and A are the contents of volatile matter and ash in the sample on a dry basis, % [35].

#### 2.2.2. Adsorption Experiments

Studies on the adsorption of heavy metal ions Pb(II), Cd(II), and Co(II) were conducted using synthetic solutions of these metal nitrates. The adsorption process was carried out using a static (batch) method. In all cases, the assumed mass of adsorbent (PWAC), $m_{a}$, was in contact with the solutions at a presumed pH and initial concentration of metal ions $C_{i}$. The process was conducted using a 358A shaker (Elpin Plus, Lubawa, Poland). The pH of the solutions was adjusted using 0.1 M NaOH or 0.1 M HCl solutions. The samples were shaken for 2 h and then left at room temperature without further shaking, maintaining contact between the adsorbent and the solution for another 22 h. The total contact time of the adsorbent with the solution was 24 h. The amount of metal adsorbed after time t ($q_{t}$) was calculated using the following formula:

(3) $$q_{t}=\frac{C_{i}\;-C_{t}}{m_{a}}V$$

where $C_{i}$ and $C_{t}$ are the initial and current (after time t) concentrations of metal ions (mg/dm^3^), respectively, V is the solution volume (dm^3^), and $m_{a}$ is the adsorbent mass (g).

#### 2.2.3. Optimization of Adsorption Conditions

To determine the best conditions for the adsorption process, one of response surface methods, namely Box–Behnken (BB) design, was used with three independent variables (factors): pH, adsorbent mass ($m_{a}$) and initial concentration of metal ions ($C_{i}$). The output variable was percentage removal (*PR*) defined as follows:

(4) $$PR=\frac{C_{i}-C_{e}}{C_{i}}\times 100\%$$

where $C_{i}$ and $C_{e}$ are the initial and equilibrium concentrations of metal ions. According to BB design, each dependent variable is coded as −1, 0 and +1, corresponding to the minimum, middle and maximum value. As a result, the three factors (pH, $m_{a}$, $C_{i}$) determine a point ($x_{1},x_{2},x_{3}$) in a three-dimensional normalized factor space. One $x_{i}$ is set to 0, whereas the two others take values ±1. This leads to 12 measurement points lying in the middle of the edges of the cube in this space. An additional point is placed in the center of the cube, where the measurement is repeated four times for higher accuracy. BB is an efficient design for estimating a quadratic model without having to perform experiments at the corners of the factor space or beyond it for extreme values on the axes as in Central Composite Design. As a result, the removal was modeled with a full quadratic function of three variables as follows:

(5) $$MPR=a_{0}+b_{1}x_{1}+b_{2}x_{2}+b_{3}x_{3}+c_{1}x_{1}^{2}+c_{2}x_{2}^{2}+c_{3}x_{3}^{2}+d_{1}x_{2}x_{3}+d_{2}x_{3}x_{1}+d_{3}x_{1}x_{2}$$

where MPR is the Modeled Percentage Removal, and 10 coefficients $a_{0}$, $b_{i}$, $c_{i}$ and $d_{i}$ are model parameters, which are determined via the least squares method. To eliminate the terms of negligible impact in MPR, the calculations were performed for all combinations of base functions 1, $x_{1}$, …., $x_{1}x_{2}$. All approximations with too-high *p*-values for the parameters were rejected. Out of the remaining models, the best one was selected based on the highest value of adjusted $R^{2}$ value, defined as follows:

(6) $$AR^{2}=1-\frac{n-1}{n-p}\left(1-R^{2}\right)$$

where n is the number of measurements (16), p is the number of used base functions (1 to 10), and $R^{2}$ is the determination coefficient.

In practice, however, $C_{i}$ is a value resulting from external conditions and not a controlled one. Therefore, the values were averaged throughout $x_{3}$ (which corresponds to $C_{i}$) and the averaged MPR was obtained as follows:

(7) $$AMPR\left(x_{1},x_{2}\right)=\frac{1}{2}\int_{-1}^{1}MPR\left(x_{1},x_{2},x_{3}\right)dx_{3}$$

Having found AMPR, it was possible to determine its maximum and overall best adsorption parameter values pH and $m_{a}$ within the considered ranges of parameters, which were as follows: pH was assumed to be in range from 4.0 to 7.0, adsorbent mass $m_{a}$ changed from 0.1 g to 0.5 g (per 0.1 L of solution), and the initial concentration $C_{i}$ was investigated in the range from 10 to 100 mg/L for Cd(II) and Co(II), and from 20 to 200 mg/L for Pb(II). In addition, correlations between PR and the initial pH, mass adsorbent, $m_{a}$, and initial concentration of metal ions, $C_{i},$ were checked to determine the overall impact of the factors on the percentage removal.

#### 2.2.4. Kinetics, Isotherms and Thermodynamics of Adsorption

Further adsorption tests were performed with adsorbent mass $m_{a}$ = 0.6 g per 0.1 L of solution and pH = 6.0. These values were established based on the optimization studies.

In the sorption kinetics studies, which aimed to assess the effect of adsorbent-solution contact time on the sorption capacity of activated carbon for the tested metal ions, the initial concentration of metal ions was 50 mg/dm^3^. The current concentrations were measured after 5, 10, 15, 30, 60, 120, and 180 min.

The adsorption isotherms were determined for concentrations of metal ions from 10 to 250 mg/L. The adsorption process was modeled using the Freundlich, Langmuir, Sips and Elovich adsorption isotherm models. These models determine the relationship between the amount of adsorbate adsorbed per unit mass of adsorbent ($q_{e}$, mg/g) and the adsorbate concentration in the solution ($C_{e}$, mg/dm^3^) in equilibrium. For this purpose, the $q_{e}$ and $C_{e}$ values were determined based on the experimental results, and then the data were fitted to the equations of individual isotherm models according to the procedures described in the literature [36,37,38]. To assess the fit quality the standard error (SE) of regression was used as follows:

(8) $$SE=\sqrt{\frac{1}{n-p}\sum_{i=1}^{n}{\left({\hat{q}}_{ei}-q_{ei}\right)}^{2}}$$

where n is the number of measurement points, p is the number of parameters in the model, and ${\hat{q}}_{ei}$ and $q_{ei}$ are modeled and measured amounts of the adsorbate adsorbed (mg/g). The obtained parameters of the models were used to assess the sorption properties of the tested material and to determine the nature of interactions occurring during the adsorption process.

The effect of temperature on the adsorption process was assessed by conducting experiments at temperatures of 20, 30, 40, and 50 °C. The studies were performed for initial concentrations of Pb(II), Cd(II), and Co(II) ions in the range of 10–250 mg/L. The thermodynamic parameters of the adsorption process, i.e., the standard change in Gibbs free energy (ΔG), enthalpy (ΔH), and entropy (ΔS), were determined based on the van’t Hoff equation:

(9) $$\ln K_{e}=\frac{\Delta S}{R}-\frac{\Delta H}{RT}$$

where R is the universal gas constant (8.314 J·mol^−1^·K^−1^), T is the absolute temperature (K), and $K_{e}$ is the unitless equilibrium constant of the adsorption process determined from Langmuir isotherm and molar mass of adsorbate, as described in [39]. The values of ΔH and ΔS were determined from the slope and intercept, respectively, of the line obtained from a plot of $\ln K_{e}$ versus $T^{-1}$. The Gibbs free energy change was determined as:

(10) ΔG=ΔH−TΔS

#### 2.2.5. Apparatus

Metal ion concentration in the samples after sorption was analyzed using an inductively coupled plasma atomic emission spectrometer (ICP-AES). pH measurements were performed using a pH/mv CP-401 pH meter (ELMETRON, Zabrze, Poland) with an accuracy of ±0.002 pH. The specific surface area and pore volume of the tested materials were determined by the nitrogen adsorption–desorption method using an ASAP 2020 Plus volumetric analyzer (Version 2.00, Micromeritics Instrument Corporation, Norcross, GA, USA). The porous structure was characterized based on low-temperature (−196 °C) nitrogen adsorption/desorption isotherms, recorded in the relative pressure range from approximately 0.01 to 0.998. Before measurements, the samples were automatically degassed at 150 °C under a vacuum of 10^−4^ mmHg. Total carbon was determined using a multi-N/C 2100 carbon analyzer (Analityk Jena, Jena, Germany) with a high-temperature furnace (HT 1300). An Analysette 22 NeXT analyzer (Fritsch, Idar-Oberstein, Germany) with a measurement range of 0.5–1500 μm was used to determine particle size distribution. The surface morphology of the obtained activated carbon before and after the heavy metal sorption process was analyzed by scanning electron microscopy (SEM) using a JSM-6610LV microscope (JEOL, Tokyo, Japan).

## 3. Results and Discussion

### 3.1. Physicochemical Characteristics of Activated Carbon

The physicochemical properties of activated carbon obtained from pyrolysis of waste plastics are presented in Table 1. Analysis of the physicochemical properties of the resulting activated carbon revealed a low moisture content (1.65%), comparable to values typical of commercial activated carbons, for which this parameter typically does not exceed 5%. The total carbon content was 45.5%, while the fixed carbon content was 39.7%, confirming the effective conversion of waste plastics into a carbonaceous material. At the same time, the volatile matter content of 22% indicates the presence of residual thermal decomposition products, which may be due to the conditions of the carbonization and activation process.

A significant feature of the tested material is the high ash content of 38.3%, which is most likely related to both the presence of inorganic additives used in plastics, including fillers and pigments, as well as the residual inorganic species originating from the K_2_CO_3_ activating agent used during the preparation of activated carbon. This value is significantly higher than the data reported in [40] for char obtained from HDPE waste, for which the ash content was only 0.16%. Furthermore, for commercial activated carbons produced from wood, coconut shells, or hard coal, the mineral content is most often in the range of 2–15% [41]. The high share of the mineral fraction in the tested material is reflected in the relatively low content of bound carbon (39.7%), which was significantly lower than the typical values for commercial activated carbons, which often exceed 70–80%. Although the inorganic fraction is likely associated primarily with additives and mineral impurities present in the waste-derived feedstock, the contribution of residual species originating from the K_2_CO_3_ activation process cannot be completely excluded, despite the post-activation washing procedure. Nevertheless, the obtained material retained a significant share of the carbon phase, indicating an effective carbonization process.

The trace element content of the tested material is presented in Table 2. Since there are no separate standards specifying the permissible content of trace elements in carbonaceous materials derived from plastic waste, the values are compared with reference values to assess the potential environmental aspects of the tested material. The analyses conducted showed that the levels of the trace elements in the tested material are lower than the permissible values for group B soils specified in the Regulation of the Minister of the Environment of 9 September 2002 [42]. In the analyzed material, the concentrations of Cd, Co, and Pb were below the limit of quantification, while for Cr, Cu, Ni, and Zn they were 14.75, 35, 4.25 and 6 mg/kg d.m., respectively. The obtained values are comparable to or lower than the concentrations reported for similar carbon materials obtained from waste materials. Hilber et al. [43] determined the contents of Cr, Cu, Ni, and Zn for biochars produced from biomass containing admixtures of plastics in the range of 40–48, 35–56, 17–22, and 129–150 mg/kg d.m., respectively, i.e., at levels higher or comparable to those obtained in this study. Furthermore, the determined concentrations of Cr, Cu, Ni, and Zn were significantly lower than the limit values specified in the European Biochar Certificate [44] for biochars intended for environmental applications. The presence of trace elements in activated carbon most likely results from contamination of the raw material, as the polymers came from municipal waste, which may contain trace amounts of metals from dyes, inks, adhesives, and technological admixtures.

**Figure 2:**
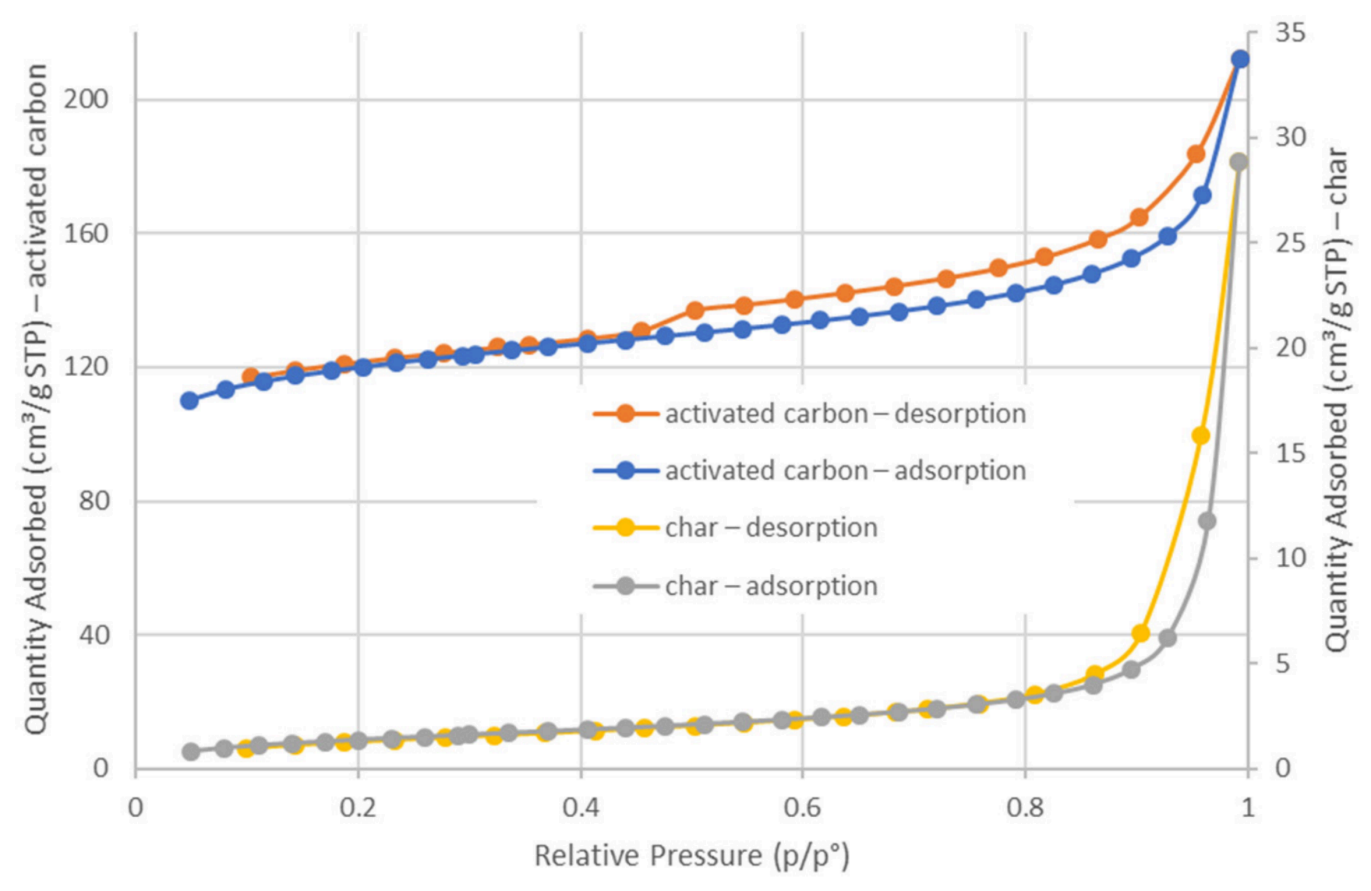
N_2_ adsorption/desorption isotherms for char and activated carbon.

**Figure 3:**
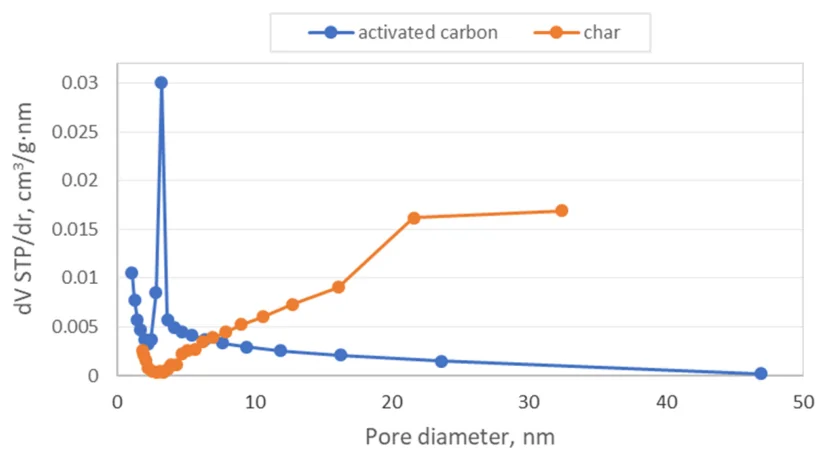
Distribution of pore volume as a function of their diameter in the char and activated carbon.

Nitrogen adsorption/desorption isotherms for the char and the activated carbon obtained from it, as well as the pore volume distribution as a function of pore diameter, are presented in Figure 2 and Figure 3, respectively. Analysis of the isotherms revealed significant differences in the textural properties of the two materials. The char was characterized by a low specific surface area and a small total pore volume, whereas the activation process resulted in a substantial development of the porous structure. According to the IUPAC classification [45], the hysteresis loops can be classified as H3 for the char and H4 for the activated carbon.

The char exhibited a relatively low nitrogen adsorption capacity and a pronounced increase in nitrogen uptake at high relative pressures (p/p^0^ > 0.9). The shape of the adsorption isotherm is close to a type II isotherm according to the IUPAC classification, indicating a material with low porosity, where adsorption occurs predominantly on the external surface and within interparticle voids. The H3 hysteresis loop is characteristic of slit-shaped voids formed between particle aggregates and does not necessarily indicate a well-developed mesoporous structure. Although the pore size distribution revealed pores with diameters of approximately 10–30 nm (Figure 3), their contribution to the total pore volume was small, which is consistent with the low specific surface area (5.3 m^2^/g) and the small total pore volume (0.045 cm^3^/g).

In contrast, the activated carbon exhibited significantly higher nitrogen uptake over the entire range of relative pressures, reflecting the substantial development of the porous structure during the activation. The steep adsorption at low relative pressures indicates the presence of a considerable fraction of micropores. Moreover, the pore size distribution (Figure 3) showed a distinct maximum at pore diameters of approximately 3–4 nm, confirming the presence of narrow mesopores. The H4 hysteresis loop characteristic is typical of micro-mesoporous materials containing small-sized slit-like pores, which are often observed in activated carbon materials [46]. This interpretation is consistent with the considerable increase in the specific surface area from 5.3 to 562 m^2^/g and the total pore volume from 0.045 to 0.329 cm^3^/g after activation. The obtained results indicate that the activation process led to a significant reconstruction of the porous structure of the char, an increase in the adsorption capacity, and an increase in the share of micropores and narrow mesopores.

Table 3 summarizes the surface textural parameters determined based on N_2_ adsorption/desorption. The determined textural parameters of the tested materials confirm that thermochemical activation of the char using K_2_CO_3_ at 800 °C resulted in significant development of the porous structure. The specific surface area increased from 5.3 to 562 m^2^/g, and the total pore volume from 0.045 to 0.329 cm^3^/g. The obtained specific surface area value is lower than for commercial activated carbons, but exceeded the values reported for some materials obtained from waste plastics, e.g., PVC (17.98 m^2^/g) [19] and contaminated post-consumer plastic mixtures (487 m^2^/g) [28]. Activated carbons produced from PET by carbonization in KOH at 700–800 °C were characterized by a significantly larger specific surface area, ranging from 625 to 1214 m^2^/g [47]. These results indicate that the specific surface area development of carbon materials derived from polymer waste depends largely on the type of raw material and the production process conditions. Activation led to the formation of both micropores (0.146 cm^3^/g) and mesopores (0.153 cm^3^/g), giving the material a hierarchical structure favorable to adsorption processes. Simultaneously, the dominant pore size decreased from approximately 24 nm to 3.2 nm (Figure 3), indicating a transition to a more uniform mesoporous structure, typical of K_2_CO_3_ chemical activation.

**Figure 4:**
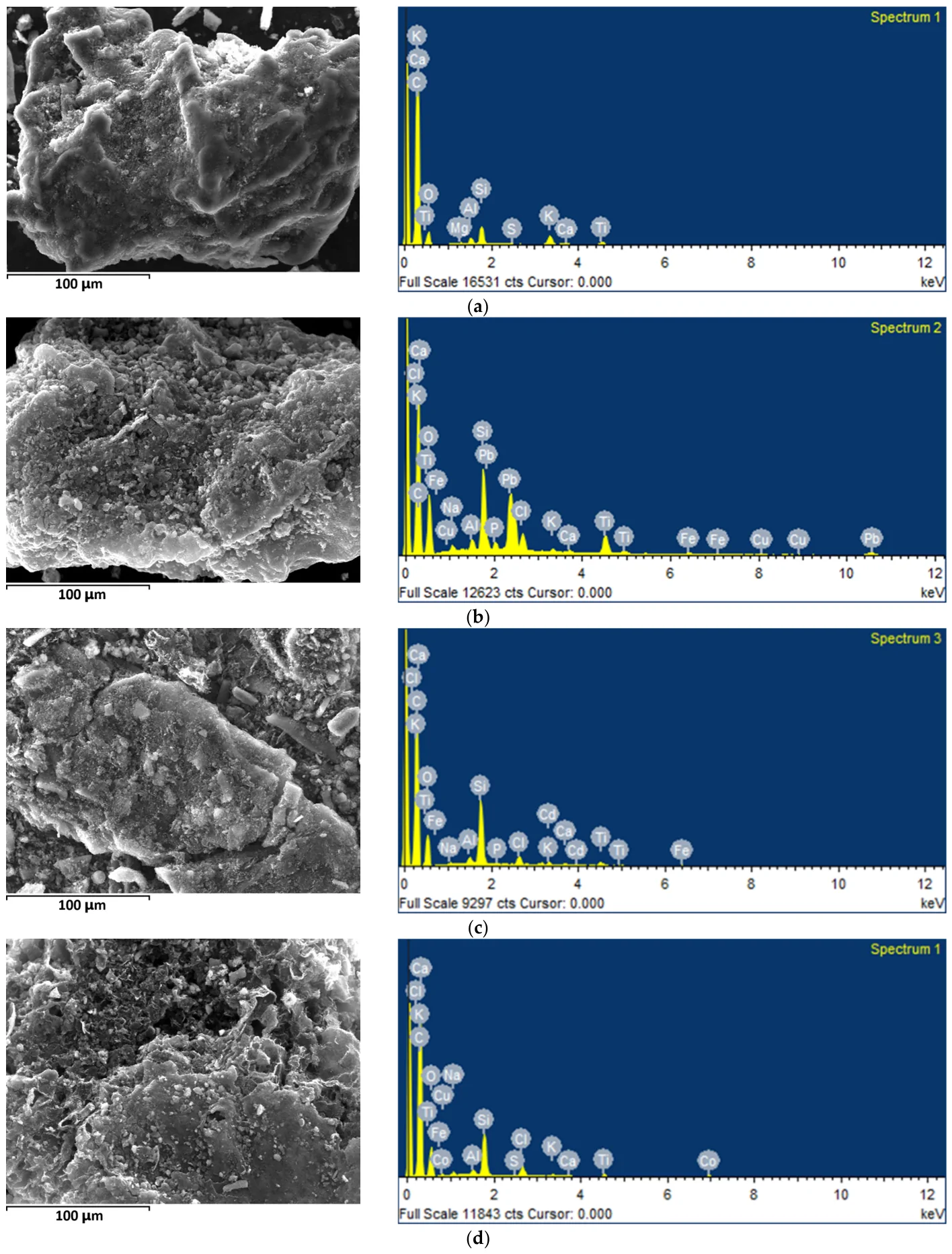
SEM images and EDS spectra of PWAC before (**a**) and after the sorption process for Pb(II) (**b**), Cd(II) (**c**) and Co(II) (**d**).

Figure 4 shows SEM micrographs and EDS energy diagrams of activated carbon before and after the Pb(II), Cd(II) and Co(II) sorption process. Morphological analysis of the obtained activated carbon revealed a well-developed, heterogeneous, and porous surface structure, characterized by numerous crevices, cavities, and irregular agglomerates (Figure 4a). SEM images also revealed the presence of fine edges, faults, and surface irregularities. These areas may act as active adsorption sites favoring the binding of heavy metal ions. EDS analysis revealed the dominance of carbon (C) and oxygen (O), as well as the presence of Ca, K, Al, Si, Mg, S, and Ti, which may originate from residues from the activated carbon production process and from additives used in plastics, such as antioxidants, mineral fillers, plasticizers, UV stabilizers, dyes, flame retardants, and impact modifiers [4].

After sorption of Pb(II), Cd(II), and Co(II) ions, SEM images (Figure 4b–d) show changes in the morphology of the adsorbent surface, including the appearance of lighter areas and local agglomerates, as well as the partial obscuration of some depressions and surface irregularities. Compared to the starting material, the surface appears less distinct and more compact, which may be attributed to the presence of adsorbed metal ions. At the same time, the overall structure of the material was preserved, with no visible signs of degradation or destruction of the activated carbon skeleton. EDS spectra confirmed the presence of Pb, Cd, and Co, respectively, in the samples after sorption, while maintaining the characteristic signals for carbon and oxygen. These results clearly indicate the effective binding of heavy metal ions by the activated carbon, while the observed morphological changes confirm the interaction between the adsorbent surface and the adsorbed ions.

**Figure 5:**
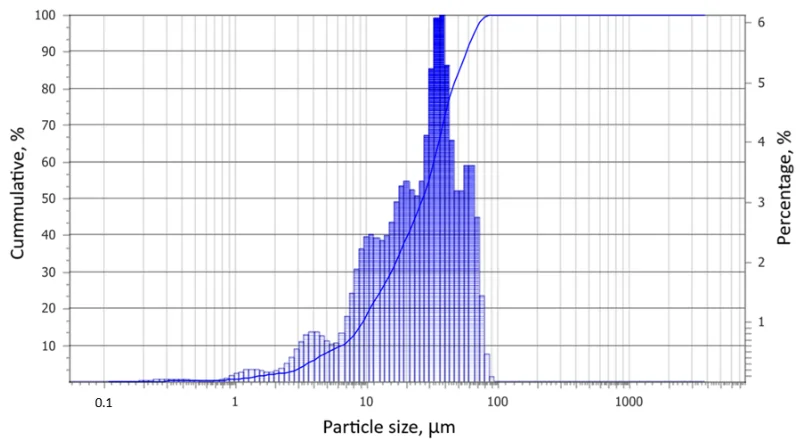
Grain size analysis of PWAC.

Granulometric analysis (Figure 5) showed that the tested activated carbon was characterized by a fine grain size and a moderately wide particle size distribution. The D10, D50, and D90 values were 6.60, 27.07, and 58.69 µm, respectively. More than half of the material (54.6%) consisted of particles smaller than 30 µm, and 84% did not exceed 50 µm. All particles had diameters below 100 µm. Fine grain size favors the development of specific surface area and increases the availability of adsorption sites, which may positively impact the efficiency of adsorption processes.

### 3.2. Optimization of Adsorption Parameters

The results of measurements according to BB design are presented in Table 4. Based on the measured equilibrium concentration, $C_{e}$, the percentage removal, PR, was calculated for each metal. The PR values fall into range 20.3–53.6%, 3.1–97.8%, and 1.6–55.6% for Cd(II), Pb(II) and Co(II), respectively. The higher removal efficiency of Pb(II) compared to Co(II)and Cd(II) may be due to the higher affinity of Pb(II) ions to the surface of activated carbon. As reported in the literature, the preferential adsorption of Pb(II) is related to the smaller hydration radius of this ion compared to other heavy metal ions, which facilitates its transport to the adsorbent surface and interaction with active sites [14,48]. The hydration radius is approximately 4.01 Å for Pb(II), 4.26 Å for Cd(II) and 4.23 Å for Co(II) [49]. This may explain the higher removal efficiency of Pb(II), despite its high initial concentration.

**Figure 6:**
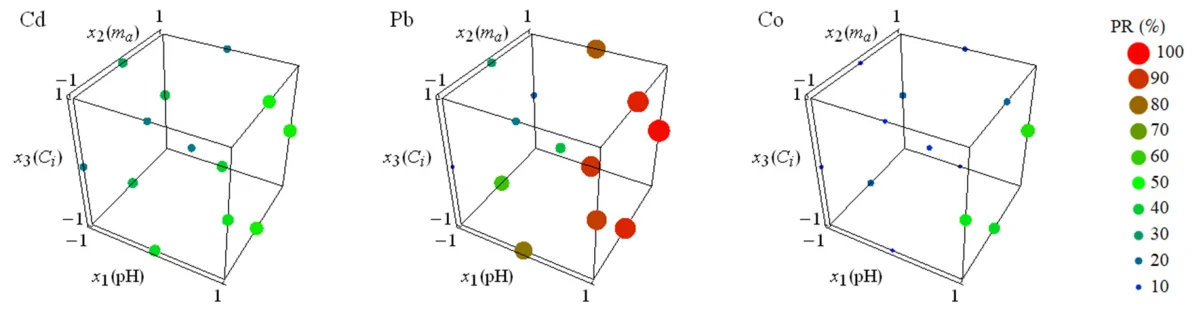
Percentage removal in BB design for Cd(II), Pb(II) and Co(II).

To better observe the results presented in Table 4, PR values are illustrated in Figure 6. It is clear that PR value grows with an increase in pH, and a *PR* reaching nearly 100% is obtained for Pb(II), whereas one of around 50% is observed for Cd(II) and Co(II).

**Figure 7:**
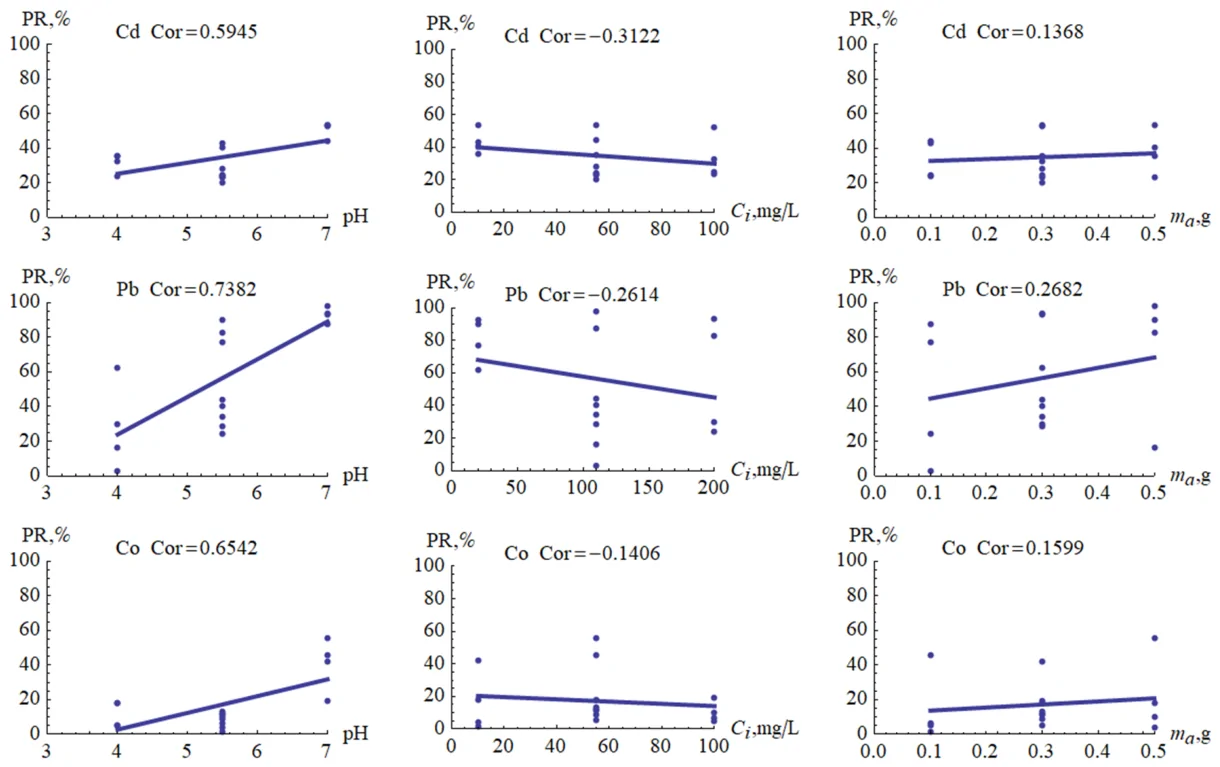
Correlations between *PR* and pH (**left column**), $C_{i}$ (**middle column**) and $m_{a}$ (**right column**) for Cd (**top row**), Pb (**middle row**) and Co (**bottom row**).

The effect of particular factors on PR value was investigated in more depth in Figure 7. In addition, correlations between PR and pH, $C_{i}$ and $m_{a}$ were shown to determine the positive or negative overall effect on PR. pH has a clearly visible positive impact on PR with correlations from 0.59 to 0.74, whereas the impact of the remaining factors is insignificant, yet negative for the initial concentration and positive for the adsorbent mass.

In the next step the MPR and AMPR functions (see Equations (5) and (7)) were constructed. The results are shown in Table 5. In all cases, the fit quality for MPR was at a similar acceptable level of around ${AR}^{2}$ ≈ 0.8. The dominating factor was solution pH—in all cases the highest MPR values were obtained for pH = 7.0. The increase in removal efficiency with increasing pH resulted primarily from reduced competition of H^+^ ions for the active sites of the adsorbent and an increase in the number of negatively charged functional groups on its surface, which favored the binding of metal cations. This phenomenon was particularly evident in the case of Pb(II), for which almost complete removal was observed at pH = 7.0 [50,51]. However, it should be noted that at this pH value, partial precipitation of Pb(OH)_2_ cannot be completely ruled out, which could have contributed to the observed high degree of removal of this metal.

The effect of adsorbent mass was noticeable only for Pb(II), with the highest MPR values for $m_{a}$ equal to 0.5 g (highest tested), whereas the adsorbent mass for Cd(II) and Co(II) has no significant effect. The initial concentration had a small negative effect, but due to averaging it in the AMPR function it was not taken into account in the final stage of optimization.

To summarize, in all cases the optimal pH was determined to be 7.0. In further tests it was lowered to 6.0 to avoid possible precipitation effects. There is also a positive impact of adsorbent mass on PR. It seems that the adsorbent mass should be slightly higher than 0.5 g, at least in the cases of Cd(II) and Co(II), for which the observed values of PR were noticeably lower than for Pb(II). Therefore, $m_{a}$ = 0.6 g (per 0.1 L) was assumed in further tests.

### 3.3. Adsorption Kinetics

**Figure 8:**
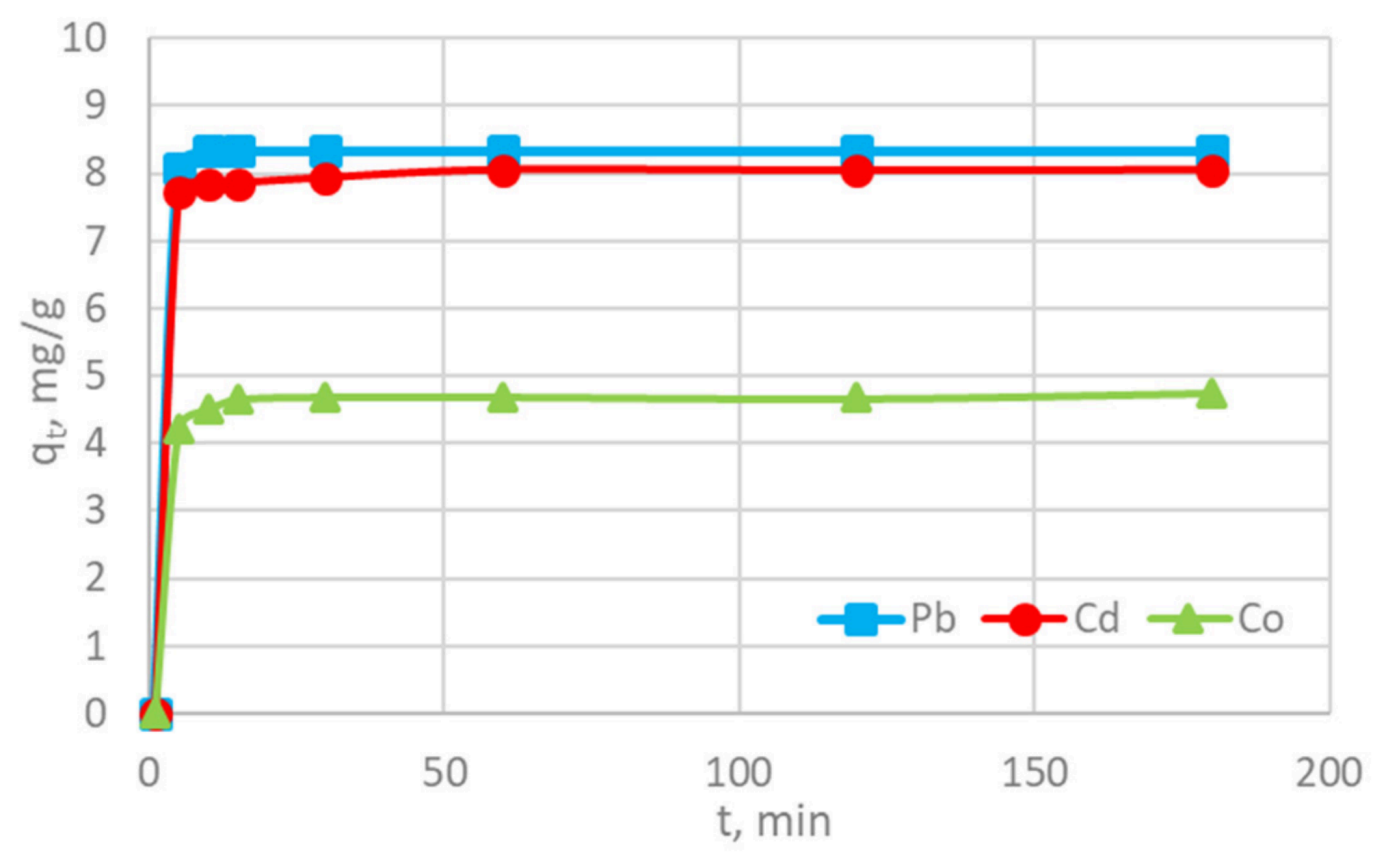
Kinetics of adsorption of Pb(II), Cd(II) and Co(II) on PWAC.

Figure 8 shows the adsorption kinetics of Pb(II), Cd(II), and Co(II) on the tested activated carbon. For all metals, the process proceeded very rapidly in the initial minutes of contact, indicating the high availability of active sites on the adsorbent surface. The highest adsorption capacity was obtained for Pb(II) ions (approx. 8.3 mg/g), with a slightly lower capacity for Cd(II) (approx. 8.0 mg/g), and the lowest for Co(II) (approx. 4.7 mg/g). The rapid increase in the q value in the first minutes of the experiment was associated with the large number of available active sites on the adsorbent surface that could bind metal ions. After approximately 15–30 min, the curves flattened, indicating that adsorption equilibrium had been reached and the active sites were saturated. The results suggest that the tested adsorbent has the highest affinity for lead ions and the lowest for cobalt ions. The equilibrium time obtained is comparable to values reported in the literature for similar adsorption systems. For example, in [19], the equilibrium time was slightly longer and amounted to 50 min for Cu(II) sorption on activated carbon obtained by thermochemical modification of polyvinyl chloride (PVC) at 700 °C in KOH.

### 3.4. Adsorption Isotherms

Based on the conducted tests the amounts of adsorbed Pb(II), Cd(II), and Co(II) ions on PWAC and their percentage removal were calculated. It follows that Pb(II) has the best sorption capacity, whereas Cd(II) has a clearly lower sorption capacity, and Co(II) has the lowest sorption capacity. At the maximum initial concentration, the adsorbed amount of Pb(II) is 35.5 mg/g, while it was 14.7 mg/g for Cd(II) and 11.6 mg/g for Co(II). The obtained sorption capacity for Cd(II) (14.7 mg/g) is similar to the value of 10.4 mg/g reported by [52] for activated carbons prepared from used tires. This demonstrates the comparable effectiveness of the tested sorbent in removing cadmium ions from aqueous solutions. Furthermore, the obtained values for Pb(II) and Cd(II) are similar to the results presented by [28] for activated carbons obtained from residues after pyrolysis of plastic waste, for which the maximum sorption capacities were approximately 40 mg/g and 13 mg/g, respectively. A similar adsorption efficiency of heavy metal ions on activated carbons obtained from pyrolysis of plastic waste was also demonstrated by [14], which confirms the high effectiveness of this type of materials in purifying aqueous solutions. The sorption capacity for Co(II) (11.6 mg/g) is comparable to the value of 13.9 mg/g obtained in [53] for activated carbon from hazelnut shells, indicating a similar mechanism for binding heavy metal ions to the surface of carbon materials. The percentage of metal ions removed from the solution depended on the type of metal and the initial concentration. This was highest for Pb(II) and ranged from 85.2% to 99.8%. Noticeably lower values were observed for Cd(II) (from 35.2% to 60.4%) and Co(II) (from 27.7% to 86.4%). At lower initial concentrations, the percentage of Co(II) sorption was higher than that for Cd(II). With increasing initial concentrations of Pb(II), Cd(II), and Co(II) ions in the solution, a decrease in their percentage sorption efficiency was observed.

**Figure 9:**
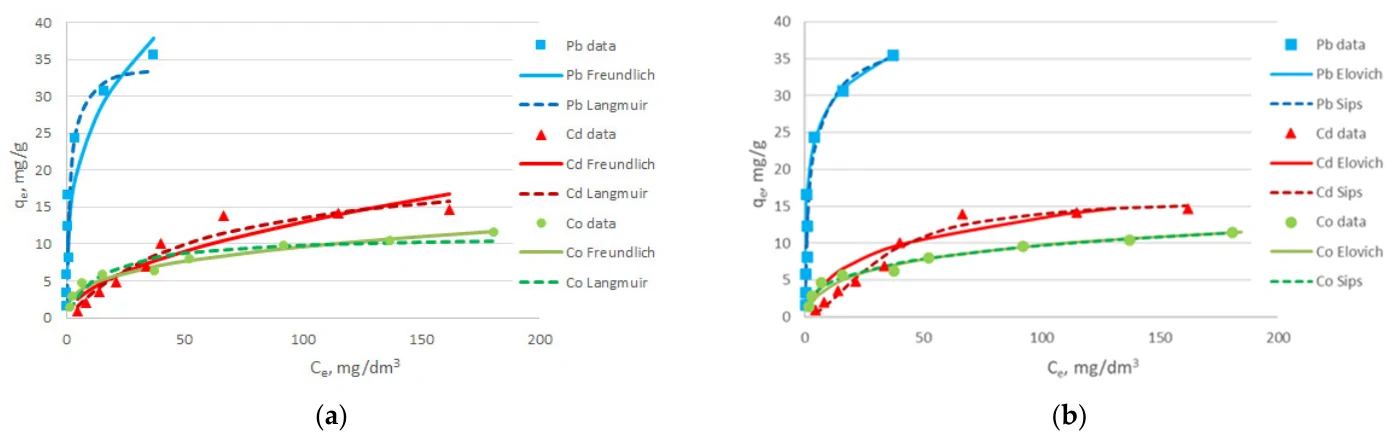
Isotherms for Pb(II), Cd(II) and Co(II) adsorption on PWAC: Langmuir and Freundlich (**a**), Elovich and Sips (**b**).

To better investigate the possible mechanisms of adsorption, the Freundlich (F), Langmuir (L), Sips (S) and Elovich (E) adsorption models were used. The results are presented in Figure 9 and Table 6.

The comparative analysis of equilibrium model performance for Pb(II), Cd(II), and Co(II) adsorption on the studied activated carbon reveals certain differences in adsorption behavior depending on the adsorbate. For Pb(II), the standard error of regression is at a similar level in all models and follows relationship S ≈ L < F < E. The small difference between the Sips and Langmuir models suggests that Pb(II) adsorption can be reasonably described by monolayer adsorption with a finite adsorption capacity. However, the superior performance of the Sips model indicates that a certain degree of surface heterogeneity is present and should be considered to achieve the most accurate description of the equilibrium data. For Cd(II), the Sips model provided the best description (S < L < F ≈ E for *SE*), confirming the combined influence of surface heterogeneity and saturation effects. In the case of Co(II), the Freundlich and Sips models showed the best and comparable performance (F ≈ S), followed by Elovich and Langmuir (F ≈ S < E < L for SE), which indicates the predominance of surface heterogeneity. The relatively better performance of Elovich for Co(II) compared to Pb(II) and Cd(II) suggests subtle differences in the distribution of adsorption energies and possibly in the interaction strength between Co(II) and surface functional groups.

**Figure 10:**
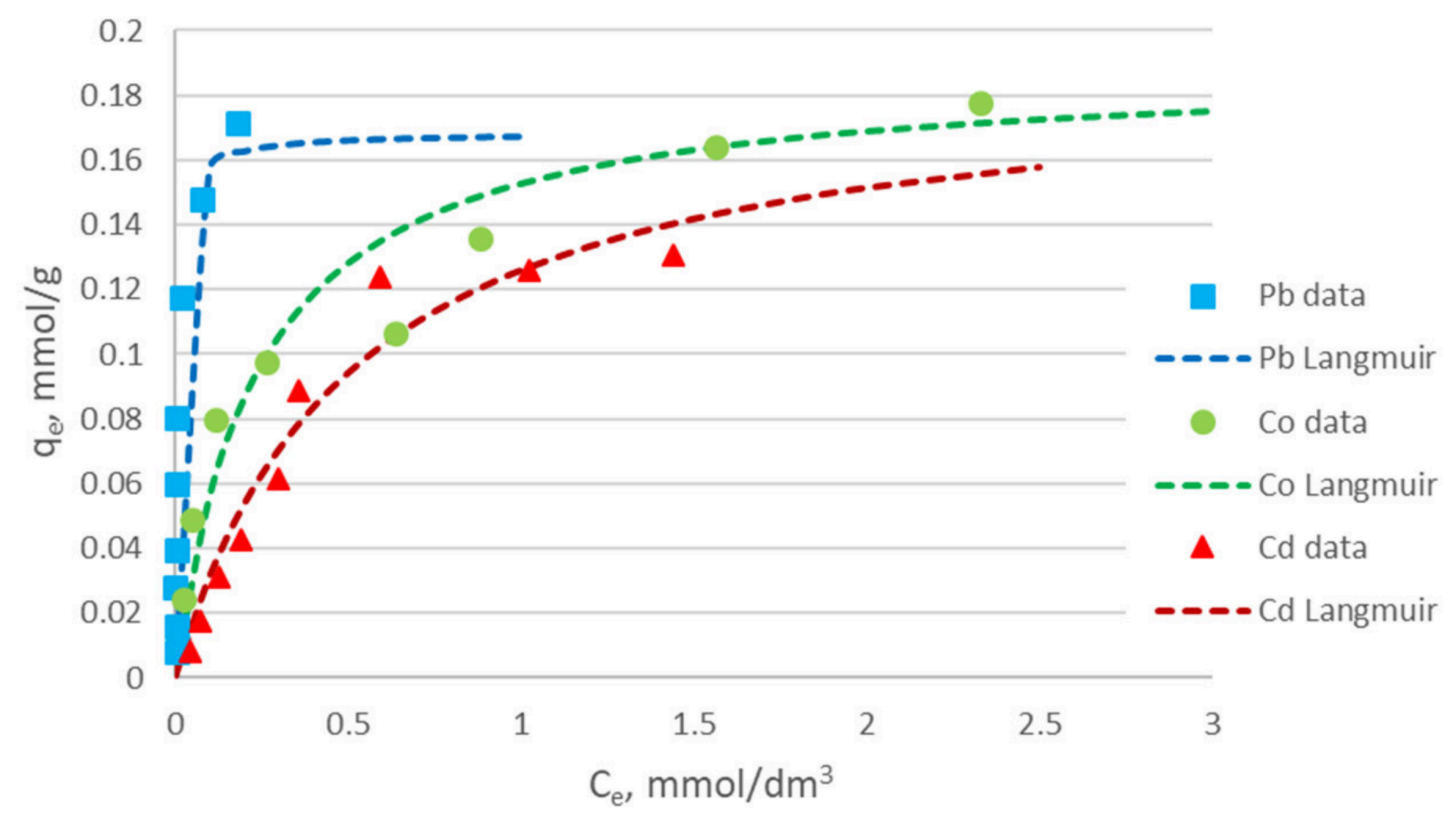
Comparison of adsorption of Pb(II), Cd(II) and Co(II) in molar units.

For further interpretation of the results, it is more convenient to use molar units, which directly indicate the number of metal ions. Figure 10 shows the measured data, with the equilibrium concentration expressed in mmol/L and the amount of adsorbed ions in mmol/g. It can be seen that the equilibrium molar concentrations for lead are too low to reliably observe saturation effects. However, using the Langmuir model, which has a simple physical interpretation, it can be seen that for all three metals, saturation occurs at a similar level, around 0.17–0.19 mmol/g (some differences can be largely attributed to measurement uncertainties). This suggests that at high concentrations, the number of adsorbed ions for each of the three metals is similar. However, this result should be treated with caution due to the low equilibrium concentrations of lead and the uncertainty regarding the corresponding isotherm. Nevertheless, the main difference lies in the region of lower equilibrium concentrations—the isotherms are arranged in the order Pb(II) > Co(II) > Cd(II). This indicates that Pb(II) is adsorbed most efficiently, then Co(II) and finally Cd(II). This relationship can be explained by the hydration radii of these metal ions (4.01 Å for Pb(II), 4.23 Å for Co(II) and 4.26 Å for Cd(II) [49])—smaller ions more easily diffuse into the pores. This suggests that the main factor influencing sorption in this case is the hydration radius of the ion, although also other physicochemical properties of the ions, such as hydration energy, ionic radius, and affinity for surface oxygen-containing functional groups, can affect the adsorption.

### 3.5. Adsorption Thermodynamics

**Figure 11:**
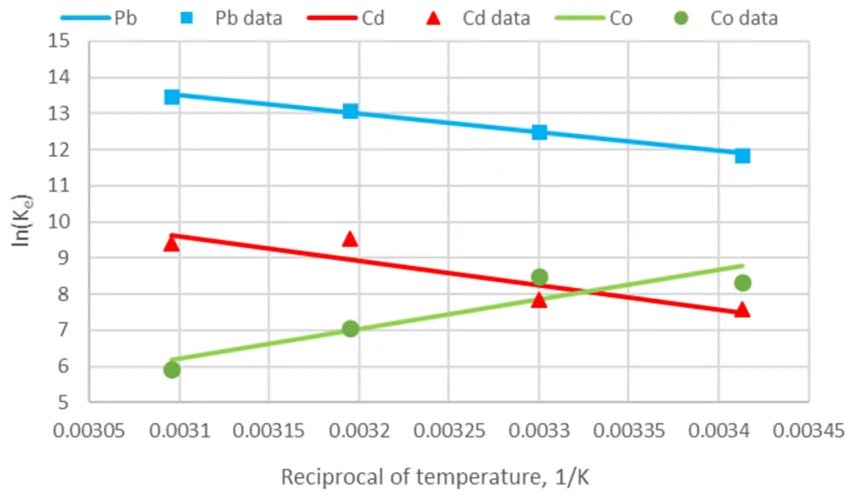
Results of thermodynamic studies of Pb(II), Cd(II) and Co(II) sorption on PWAC.

The analysis results are presented in Table 7 and Figure 11. The negative values of Gibbs free energy change (ΔG) for all metal ions tested indicate that the sorption process is spontaneous. For Pb(II) and Cd(II), the ΔG values become more negative with increasing temperature, indicating that sorption of these ions is increasingly favorable. This is also confirmed by the van’t Hoff plots (Figure 11), which show an increase in $\ln K_{e}$ values with increasing temperature. The positive values of sorption enthalpy change (ΔH) for Pb(II) (42.8 kJ/mol) and Cd(II) (56.4 kJ/mol) indicate the endothermic nature of the process. In the case of Co(II), a different process course was observed—with increasing temperature, the ΔG values became less negative, indicating a decrease in the spontaneity of sorption. This is confirmed by the negative enthalpy value (ΔH = −67.7 kJ/mol), indicating the exothermic nature of the process. This means that Co(II) sorption is more favorable at lower temperatures. Positive entropy values for Pb(II) and Cd(II) indicate an increase in disorder at the sorbent–solution interface during sorption. In contrast, the negative ΔS value for Co(II) (−0.16 kJ/(mol K)) shows an increase in the order of the system, which may be related to the stronger interaction of cobalt ions with the sorbent surface. The sorption enthalpy values obtained for Pb(II), Cd(II), and Co(II) exceed the range characteristic for physical adsorption ($\left|\Delta H\right|$ < 40 kJ/mol), suggesting a contribution of specific chemical interactions to the sorption process. This finding may be related to the heterogeneous surface of the investigated activated carbon and the presence of oxygen-containing functional groups capable of participating in ion exchange and surface complexation with metal ions [51]. In addition, the relatively high ash content of the material (38.3%) indicates a substantial contribution of inorganic components, which may also provide active sites for specific interactions with Pb(II), Cd(II), and Co(II). Thus, the removal of metal ions may involve a combination of physical adsorption, ion exchange, and surface complexation.

### 3.6. Comparative Analysis

The properties of the obtained activated carbon were compared with the literature data summarized in Table 8. The available literature data on heavy metal adsorption on activated carbons derived solely from waste plastics are still relatively sparse, with most studies focusing on adsorbents derived from biomass or hybrid materials. Against this backdrop, the tested adsorbent demonstrated sorption capacities comparable to, and in the case of Pb(II), even higher than, those reported for other adsorbents derived from post-consumer plastic waste. The obtained results confirm that waste plastics are a valuable raw material for obtaining activated carbons with good sorption properties, which can be used in heavy metal removal processes from water and wastewater and as materials limiting the mobility of heavy metals in contaminated soils.

## 4. Conclusions

Based on the results obtained in the research, the following conclusions can be formulated:

- Post-pyrolysis char from waste plastics can be a valuable raw material for the production of carbon-based adsorbents. The benefit may be twofold—the management and processing of plastic waste and its use for the adsorption of harmful substances such as heavy metal ions.
- The activated post-pyrolysis char exhibits typical characteristics of activated carbons, but with a moderate specific surface area (~562 m^2^/g) lower than that of commercial materials. This may be attributed to the relatively high ash content, as well as partial pore blockage by residual potassium species and incomplete development of microporosity. Nevertheless, the presence of a balanced proportion of micro- and mesopores makes the material potentially useful for adsorption in both the gas and liquid phases.
- The obtained material showed good adsorptive properties with respect to Pb(II), Cd(II) and Co(II). The adsorption capacity reached 35.5 mg/g for Pb(II), 14.7 mg/g for Cd(II), and 11.6 mg/g for Co(II). The adsorption process was rapid.
- Energy distribution and adsorption mechanism depend on metal ion, and reflect differences in hydration radii, and probably also hydration energy, ionic radius, and affinity toward oxygen-containing surface functional groups.
- Thermodynamic analysis showed that the sorption of Pb(II), Cd(II), and Co(II) on the obtained activated carbon occurs spontaneously. An increase in temperature favors the sorption of Pb(II) and Cd(II) (endothermic process), while reducing the efficiency of Co(II) sorption, which is exothermic.

Further research should be primarily focused on reducing the mineral content, which could contribute to increasing the percentage of bound carbon and improving the adsorption properties of the resulting activated carbon. The second important issue is the regeneration of the obtained activated carbon.

## Figures and Tables

**Table 1:** Physicochemical characteristics of activated carbon obtained from pyrolysis of waste plastics.

Quantity	Moisture Content (%)	Ignition Loss (%)	Ash (%)	Volatile Matter (%)	Total Carbon (wt%)	Fixed Carbon (%)
**Value**	1.65 ± 0.055	61.7 ± 0.2	38.3 ± 0.3	22.0 ± 0.08	45.5	39.7

**Table 2:** Content of trace elements in the tested material and the reference values (in mg/kg d.m.).

Element	Cd	Co	Cr	Cu	Ni	Pb	Zn
Content in PWAC	<0.01	<0.01	14.75	35	4.25	<0.01	6
Permissible values in group B soils [42]	4	20	150	150	100	100	300
Content reported in [43]	<0.2	–	40–48	35–56	17–22	14–20	129–150
Limit values by EBC [44]	0.7–1.5	–	70–90	70–100	25–50	10–120	200–400

**Table 3:** Textural parameters of the char and activated carbon.

Quantity	Unit	Value	
		Char	PWAC
Specific surface area	m^2^/g	5.3	562
Total pore volume	cm^3^/g	0.045	0.329
Micropore volume by t-Plot	cm^3^/g	–	0.146
Pore size range by BJH desorption	nm	1.7–50	0.5–300
Cumulative pore volume by BJH desorption	cm^3^/g	0.023	0.153
Cumulative pore area by BJH desorption	m^2^/g	4.78	78.4
Dominant mesopore size by BJH desorption	nm	24	3.2

**Table 4:** BB design and measurement results for adsorption optimization.

Factor Value			Coded Factor Value			Final Concentration			Percentage Removal		
pH	$m_{a}$	$C_{i}$ *	$x_{1}$	$x_{2}$	$x_{3}$	*C_e_* (mg/L)			PR (%)		
–	g	mg/L	–	–	–	Cd	Pb	Co	Cd	Pb	Co
4.0	0.1	55	−1	−1	0	42.0	106.6	52.0	23.6	3.1	5.4
4.0	0.3	10	−1	0	−1	6.4	7.6	8.2	35.6	62.2	17.9
4.0	0.3	100	−1	0	1	67.6	140.3	95.0	32.4	29.8	5.0
4.0	0.5	55	−1	1	0	35.5	92.3	45.0	35.5	16.1	18.1
5.5	0.1	10	0	−1	−1	5.7	4.6	9.8	42.8	77.2	1.6
5.5	0.1	100	0	−1	1	75.5	151.9	93.5	24.5	24.1	6.5
5.5	0.3	55	0	0	0	43.8	61.6	47.8	20.3	44.0	13.0
5.5	0.3	55	0	0	0	41.6	72.4	50.2	24.4	34.2	8.7
5.5	0.3	55	0	0	0	39.5	78.6	48.8	28.2	28.6	11.3
5.5	0.3	55	0	0	0	42.4	65.7	48.4	23.0	40.3	12.0
5.5	0.5	10	0	1	−1	5.9	2.0	9.6	40.7	90.3	3.9
5.5	0.5	100	0	1	1	76.8	34.8	90.2	23.2	82.6	9.8
7.0	0.1	55	1	−1	0	30.7	13.5	30.0	44.3	87.7	45.6
7.0	0.3	10	1	0	−1	4.6	1.4	5.8	53.6	92.9	42.0
7.0	0.3	100	1	0	1	47.6	13.0	80.8	52.4	93.5	19.2
7.0	0.5	55	1	1	0	25.6	2.4	24.4	53.4	97.8	55.6
								min	20.3	3.1	1.6
								max	53.6	97.8	55.6

* the values in this column are $C_{i}$ for Co and Cd; the values of $C_{i}$ for Pb are twice as large.

**Table 5:** Optimization results for adsorption parameters.

Metal Ions	Quantity	Value
Cd(II)	MPR	$25.1+9.6x_{1}+13.0x_{1}^{2}-5.0x_{3}+6.6x_{3}^{2}$
	${AR}^{2}$	0.81
	AMPR	$27.0+0.6x_{1}+13.0x_{1}^{2}$
	optimum	$x_{1}=1\;\;\;\;\;(\mathrm{pH}=7.0)$
Pb(II)	MPR	$44.0+32.6x_{1}+11.8x_{2}-11.5x_{3}+25.1x_{3}^{2}$
	${AR}^{2}$	0.79
	AMPR	$52.3+32.6x_{1}+11.8x_{2}$
	optimum	$x_{1}=1,\;\;\;x_{2}=1\;\;\;\;\;\;\left(\mathrm{pH}=7.0,\;\;\;\;m_{a}=0.5\;g\right)$
Co(II)	MPR	$12.3+14.5x_{1}+17.7x_{1}^{2}-8.0x_{3}^{2}$
	${AR}^{2}$	0.77
	AMPR	$9.8+14.5x_{1}+17.7x_{1}^{2}$
	optimum	$x_{1}=1\;\;\;\;\;\;\left(\mathrm{pH}=7.0\right)$

**Table 6:** Parameters of isotherm models for the sorption of Pb(II), Cd(II) and Co(II) on PWAC.

Parameter or Indicator	Unit	Pb(II)	Cd(II)	Co(II)
Freundlich (F) isotherm: ${\hat{q}}_{e}=K_{F}C_{e}^{1/n_{F}}$				
*K*_F_	mg/g (L/mg)^1/*n*F^	12.7	1.13	2.13
*n*_F_	–	3.31	1.89	3.06
*SE*	mg/g	4.5	2.0	0.6
Langmuir (L) isotherm: ${\hat{q}}_{e}=Q_{L}K_{L}C_{e}/(1+K_{L}C_{e})$				
*K*_L_	L/mg	0.675	0.0175	0.0713
*Q*_L_	mg/g	34.8	21.4	11.1
*SE*	mg/g	4.3	1.3	1.1
Sips (S) isotherm: ${\hat{q}}_{e}=Q_{S}{\left(K_{S}C_{e}\right)}^{m_{S}}/\left(1+{\left(K_{S}C_{e}\right)}^{m_{S}}\right)$				
*K*_S_	L/mg	0.477	0.0310	0.00174
*Q*_S_	mg/g	42.3	16.0	30.1
*m*_S_	–	0.651	1.74	0.426
*SE*	mg/g	4.2	1.0	0.6
Elovich (E) isotherm: ${\hat{q}}_{e}=K_{E}Q_{E}C_{e}\exp\left(-{\hat{q}}_{e}/Q_{E}\right)$				
*K_E_*	L/mg	33.0	0.173	0.264
*Q*_E_	mg/g	6.55	6.43	4.07
*SE*	mg/g	5.0	2.2	0.8

SE—standard error of regression.

**Table 7:** Thermodynamic parameters of Pb(II), Cd(II) and Co(II) sorption on PWAC.

Temperature, T [°C]	$K_{L}$ [L/mg]			The Gibbs Free Energy Change (Δ*G*) [kJ/mol]		
	Pb(II)	Cd(II)	Co(II)	Pb(II)	Cd(II)	Co(II)
20	0.675	0.0175	0.0713	−29.0	−18.2	−21.4
30	1.29	0.0223	0.0831	−31.4	−20.8	−19.8
40	2.33	0.123	0.0201	−33.9	−23.3	−18.2
50	3.39	0.107	0.00631	−36.3	−25.9	−16.6
ΔH [kJ/mol]	42.8 ± 2.7	56.4 ± 18.8	−67.7 ± 20.5			
ΔS [kJ/(mol·K)]	0.24 ± 0.01	0.26 ± 0.06	−0.16 ± 0.07			
*R* ^2^	0.992	0.818	0.845			

**Table 8:** Comparison of the tested activated carbon with selected adsorbents from plastic waste, biomass and hybrid materials.

Adsorbent Precursor	Method of Production	Metal	Maximum Sorption Capacity (mg/g)	Reference
Municipal waste (polyolefin fraction of packaging waste)	pyrolysis 800 °C activation K_2_CO_3_	Cd(II) Co(II) Pb(II)	14.7 11.6 35.5	This study
Post-consumer mixed plastics	pyrolysis 800 °C activation Na_2_CO_3_	Cd(II) Cu(II) Pb(II)	13 12 40	[14]
Mixed post-consumer plastic waste (PP, PS, and PE)	pyrolysis 550 °C	Pb(II)	26	[54]
PET + shrimp waste	co-pyrolysis 800 °C activation KOH	Cd(II)	22.3	[55]
Worn tires	pyrolysis 600 °C activation KOH	Pb(II)	93.18	[56]
Worn tires	pyrolysis 900 °C activation H_2_O_2_ 500 °C	Cu(II) Pb(II) Zn(II) (multi-metal system)	12.44 9.68 5.0	[57]
Straw + PE film (agricultural film)	co-pyrolysis 600 °C	Pb(II)	192.2	[58]
Poplar leaves + brown coal	co-pyrolysis 700 °C activation KOH modification Fe_3_O_4_	Cd(II) Pb(II)	4.93 10.55	[59]
Tobacco stalks	pyrolysis 800 °C activation KOH	Cd(II) Pb(II) Zn(II)	28.7 60 32	[60]
Coconut shells	pyrolysis 500 °C activation H_3_PO_4_	Pb(II)	49.92	[61]

## Data Availability

The original contributions presented in this study are included in the article. Further inquiries can be directed to the corresponding author.

## Abbreviations

The following abbreviations are used in this manuscript:

BB	Box–Behnken design
BJH	Barrett–Joyner–Halenda
EDS	Energy-Dispersive X-ray Spectroscopy
PE	Polyethylene
PET	Poly(Ethylene Terephthalate)
PP	Polypropylene
PWAC	Polymer Waste-based Activated Carbon
SEM	Scanning Electron Microscopy

## References

[1] Plastics Europe (2025). Tworzywa—Fakty 2025 w Pigułce. Światowa i Europejska Produkcja Tworzyw Sztucznych Oraz Wybrane Wskaźniki Ekonomiczne.

[2] Jabłońska B., Poznańska G., Jabłoński P., Dziadas M. (2025). Thermochemical conversion of mixed plastics from car dismantling by pyrolysis and distillation and potential applications of the products. Waste Manag..

[3] Hahladakis J.N., Velis C.A., Weber R., Iacovidou E., Purnell P. (2018). An overview of chemical additives present in plastics: Migration, release, fate and environmental impact during their use, disposal and recycling. J. Hazard. Mater..

[4] Carrot C., Bendaoud A., Pillon C., Olabisi O., Adewale K. (2016). Polyvinyl Butyral. Handbook of Thermoplastics.

[5] Harussani M.M., Sapuan S.M., Rashid U., Khalina A., Ilyas R.A. (2022). Pyrolysis of polypropylene plastic waste into carbonaceous char: Priority of plastic waste management amidst COVID-19 pandemic. Sci. Total Environ..

[6] Hashemi M., Nguyen K.T.Q., Robert D.J., Zhang G.K., Hosseinnejad T., Marney D. (2026). Advanced pyrolysis methods for mixed plastic waste management: Enhanced char and gas yields. J. Anal. Appl. Pyrolysis.

[7] Jabłońska B., Poznańska G., Jabłoński P., Zwolińska J. (2024). Thermochemical Valorization of Plastic Waste Containing Low Density Polyethylene, Polyvinyl Chloride and Polyvinyl Butyral into Thermal and Fuel Energy. Energies.

[8] Worrel W.A., Vesilind P.A. (2012). Solid Waste Engineering.

[9] van Akker M., Strien J.R.J., Genuino H.C., van Eijk M.C.P., Schenk N.J., Heeres H.J., Deuss P.J. (2025). Full utilization of hard-to-recycle mixed plastic waste by conversion toward pyrolysis oil and BTX aromatics on a pilot scale. Energy Fuels.

[10] Poznańska G., Jabłońska B., Zawieja I. (2024). Możliwości zastosowania karbonizatu pozyskanego z pirolizy odpadowych tworzyw sztucznych. Nowatorskie Rozwiązania w Inżynierii Środowiska i Energetyce-Perspektywa Zrównoważonego Rozwoju.

[11] Cai W., Liu P., Chen B., Xu H., Liu Z., Zhou Q., Yu F., Liu M., Chen M., Liu J. (2019). Plastic waste fuelled solid oxide fuel cell system for power and carbon nanotube cogeneration. Int. J. Hydrogen Energy.

[12] Zaman C.Z., Pal K., Yehye W.A., Sagadevan S., Shah S.T., Adebisi G.A., Marliana E., Rafique R.F., Johan R.B., Samer M. (2017). Pyrolysis: A sustainable way to generate energy from waste. Pyrolysis.

[13] Harussani M.M., Sapuan S.M., Khalina A., Rashid U., Tarique J. Slow pyrolysis of disinfected COVID-19 non-woven polypropylene (PP) waste. Proceedings of the International Symposium on Applied Sciences and Engineering (ISASE 2021).

[14] Pereira L., Castillo V., Calero M., Blázquez G., Solís R.R., Martín-Lara M. (2024). Conversion of char from pyrolysis of plastic wastes into alternative activated carbons for heavy metal removal. Environ. Res..

[15] Poznańska G., Jabłońska B., Jabłoński P., Piotrowski T. (2026). The Influence of Heterogeneity of Polyolefin Waste and Alu-PEX Laminates on the Composition and Yield of Pyrolysis Gas: A Comparative Analysis with RDF. Energies.

[16] Adeniyi A.G., Iwuozor K.O., Emenike E.C., Ajala O.J., Ogunniyi S., Muritala K.B. (2024). Thermochemical co-conversion of biomass-plastic waste to biochar: A review. Green Chem. Eng..

[17] Elnour A.Y., Alghyamah A.A., Shaikh H.M., Poulose A.M., Al-Zahrani S.M., Anis A., Al-Wabel M.I. (2019). Effect of Pyrolysis Temperature on Biochar Microstructural Evolution, Physicochemical Characteristics, and Its Influence on Biochar/Polypropylene Composites. Appl. Sci..

[18] Bhattacharya R. (2023). A review on production and application of activated carbon from discarded plastics in the context of ‘waste treats waste’. J. Environ. Manag..

[19] Ziane F., Amokrane S., Murillo R., Ouassel S., Nibou D. (2022). Recovery of synthetic copper ions by activated carbon from an industrial plastic PVC waste: Equilibrium, dynamic, kinetic and thermodynamic studies. Chem. Phys. Lett..

[20] Qasem N.A.A., Mohammed R.H., Lawal D.U. (2021). Removal of heavy metal ions from wastewater: A comprehensive and critical review. npj Clean Water.

[21] Oladimeji T.E., Oyedemi M., Emetere M.E., Agboola O., Adeoye J.B., Odunlami O.A. (2024). Review on the impact of heavy metals from industrial wastewater effluent and removal technologies. Heliyon.

[22] Nnaji N.D., Onyeaka H., Miri T., Ugwa C. (2023). Bioaccumulation for heavy metal removal: A review. SN Appl. Sci..

[23] Oladimeji T.E., Oyedemi M.O., Odunfa M.K., Agboola A., Adeoye J.B., Oke M.A., Akindele O.O. (2025). From origin to oversight: Properties, impacts and management of heavy metals. Discov. Appl. Sci..

[24] Kumar M., Singh S., Jain A., Yadav S., Dubey A., Trivedi S.P. (2024). A review on heavy metal-induced toxicity in fishes: Bioaccumulation, antioxidant defense system, histopathological manifestations, and transcriptional profiling of genes. J. Trace Elem. Med. Biol..

[25] Lai Y., Gao F., Ge R., Liu R., Ma S., Liu X. (2024). Metal ions overloading and cell death. Cell Biol. Toxicol..

[26] Stubblefield W.A., Van Genderen E., Cardwell A.S., Heijerick D.G., Janssen C.R., De Schamphelaere K.A.C. (2020). Acute and Chronic Toxicity of Cobalt to Freshwater Organisms: Using a Species Sensitivity Distribution Approach to Establish International Water Quality Standards. Environ. Toxicol. Chem..

[27] Blanchard R., Mekonnen T.H. (2024). Valorization of plastic waste via chemical activation and carbonization into activated carbon for functional material applications. RSC Appl. Polym..

[28] Ligero A., Calero M., Pérez A., Solís R.R., Muñoz-Batista M.J., Martín-Lara M.A. (2023). Low-cost activated carbon from the pyrolysis of post-consumer plastic waste and its application in CO_2_ capture. Process Saf. Environ. Prot..

[29] Ajiboye T.O., Oyewo O.A., Onwudiwe D.C. (2021). Simultaneous removal of organics and heavy metals from industrial wastewater: A review. Chemosphere.

[30] Feng J., Burke I.T., Chen X., Steward D.I. (2023). Assessing metal contamination and speciation in sewage sludge: Implications for soil application and environmental risk. Rev. Environ. Sci. Biotechnol..

[31] Shrestha R., Ban S., Devkota S., Sharma S., Joshi R., Tiwari A.P., Kim H.Y., Joshi M.K. (2021). Technological trends in heavy metals removal from industrial wastewater: A review. J. Environ. Chem. Eng..

[32] (2017). Biopaliwa Stałe—Oznaczanie Zawartości Wilgoci—Metoda Suszarkowa—Część 2: Wilgotność Całkowita—Metoda Uproszczona.

[33] (2002). Stałe Paliwa Mineralne—Oznaczanie Popiołu.

[34] (2016). Biopaliwa Stałe—Oznaczanie Zawartości Części Lotnych.

[35] Ramirez A., Ocampo R., Giraldo S., Padilla E., Flórez E., Acelas N. (2020). Removal of Cr(VI) from an aqueous solution using an activated carbon obtained from teakwood sawdust: Kinetics, equilibrium, and density functional theory calculations. J. Environ. Chem. Eng..

[36] Hamdaoui O., Naffrechoux E. (2007). Modeling of adsorption isotherms of phenol and chlorophenols onto granular activated carbon. Part I. Two-parameter models and equations allowing determination of thermodynamic parameters. J. Hazard. Mater..

[37] Hamdaoui O., Naffrechoux E. (2007). Modeling of adsorption isotherms of phenol and chlorophenols onto granular activated carbon. Part II. Models with more than two parameters. J. Hazard. Mater..

[38] Jabłońska B. (2020). Removing of Cr(III) and Cr(VI) compounds from aqueous solutions by shale waste rocks. Desalin. Water Treat..

[39] Ghosal P.S., Gupta A.K. (2017). Determination of thermodynamic parameters from Langmuir isotherm constant-revisited. J. Mol. Liq..

[40] Jamradloedluk J., Lertsatitthanakorn C. (2014). Characterization and utilization of char derived from fast pyrolysis of plastic wastes. Procedia Eng..

[41] Ganjoo R., Sharma S., Kumar A., Daouda M.M.A., Verma C., Quraishi M.A. (2023). Activated Carbon: Fundamentals and Applications. Activated Carbon Progress and Applications.

[42] (2002). Rozporządzenie Ministra Środowiska z Dnia 9 Września 2002 r. w Sprawie Standardów Jakości Gleby Oraz Standardów Jakości Ziemi. Dz. U..

[43] Hilber I., Hagemann N., de la Rosa J., Knicker H., Bucheli T., Schmidt H.-P. (2024). Biochar Production from Plastic-Contaminated Biomass. GCB Bioenergy.

[44] European Biochar Certificate (EBC) (2023). European Biochar Certificate—Guidelines for a Sustainable Production of Biochar.

[45] Thommes M., Kaneko K., Neimark A.V., Olivier J.P., Rodriguez-Reinoso F., Rouquerol J., Sing K.S.W. (2015). Physisorption of gases, with special reference to the evaluation of surface area and pore size distribution (IUPAC Technical Report). Pure Appl. Chem..

[46] Serafin J., Dziejarski B. (2024). Activated carbons—Preparation, characterization and their application in CO_2_ capture: A review. Environ. Sci. Pollut. Res..

[47] Wang Z., Lin T., Chen W. (2020). Occurrence and removal of microplastics in an advanced drinking water treatment plant (ADWTP). Sci. Total Environ..

[48] Mobasherpour I., Salahi E., Pazouki M. (2012). Comparative of the removal of Pb^2+^, Cd^2+^ and Ni^2+^ by nano crystallite hydroxyapatite from aqueous solutions: Adsorption isotherm study. Arab. J. Chem..

[49] Nightingale E.R. (1959). Phenomenological Theory of Ion Solvation. Effective Radii of Hydrated Ions. J. Phys. Chem..

[50] Lu H., Zhang W., Yang Y., Huang X., Wang S., Qiu R. (2012). Relative distribution of Pb^2+^ sorption mechanisms by sludge-derived biochar. Water Res..

[51] Yang X., Wan Y., Zheng Y., He F., Yu Z., Huang J., Wang H., Ok Y.S., Jiang Y., Gao B. (2019). Surface functional groups of carbon-based adsorbents and their roles in the removal of heavy metals from aqueous solutions: A critical review. Chem. Eng. J..

[52] Nieto-Márquez A., Pinedo-Flores A., Picasso G., Atanes E., Sun Kou R. (2017). Selective adsorption of Pb^2+^, Cr^3+^ and Cd^2+^ mixtures on activated carbons prepared from waste tires. J. Environ. Chem. Eng..

[53] Demirbaş E. (2003). Adsorption of Cobalt(II) Ions from Aqueous Solution onto Activated Carbon Prepared from Hazelnut Shells. Adsorpt. Sci. Technol..

[54] Martín-Lara M.A., Piñar A., Ligero A., Blázquez G., Calero M. (2021). Characterization and Use of Char Produced from Pyrolysis of Post-Consumer Mixed Plastic Waste. Water.

[55] Aljomard H., Abushawish A., Almanassra I.W., Manasrah A.D., Atieh M.A., Mohammad A.W., Hamzah S. (2026). Batch and fixed-bed adsorption of Cd(II) onto hierarchically porous carbon co-valorized from shrimp and plastic waste: Breakthrough behavior, modeling, and mechanistic insights. Diam. Relat. Mater..

[56] Rutto H., Seidigeng T., Malise L. (2019). Adsorption of lead ions onto chemically activated carbon from waste tire char and optimization of the process using response surface methodology. Arch. Environ. Prot..

[57] Cherono F., Mburu N., Kakoi B. (2021). Adsorption of lead, copper and zinc in a multi-metal aqueous solution by waste rubber tires for the design of single batch adsorber. Heliyon.

[58] Sun B., Xu X., Zhang Z., Zhang C., Jiao Y., Li T., Wang S., Wang S., Li Z. (2026). Performance regulation and adsorption behavior of carbon-based adsorbent prepared through co-pyrolysis of straw and agricultural film. J. Anal. Appl. Pyrolysis.

[59] Zhang X., Li Y., He Y., Kong D., Klein B., Yin S., Zhao H. (2022). Preparation of Magnetic Activated Carbon by Activation and Modification of Char Derived from Co-Pyrolysis of Lignite and Biomass and Its Adsorption of Heavy-Metal-Containing Wastewater. Minerals.

[60] Jabłońska B., Jabłoński P., Gęga J. (2025). Kinetics and Thermodynamics of Pb(II), Zn(II), and Cd(II) Adsorption from Aqueous Solutions onto Activated Biochar Obtained from Tobacco Waste. Materials.

[61] Sharaf El-Deen G.E., Sharaf El-Deen S.E.A. (2016). Kinetic and isotherm studies for adsorption of Pb(II) from aqueous solution onto coconut shell activated carbon. Desalin. Water Treat..

